# NGL Viewer: a web application for molecular visualization

**DOI:** 10.1093/nar/gkv402

**Published:** 2015-04-29

**Authors:** Alexander S. Rose, Peter W. Hildebrand

**Affiliations:** Institut für Medizinische Physik und Biophysik, AG ProteInformatics, Charité–Universitätsmedizin Berlin, Charitéplatz 1, 10117 Berlin, Germany

## Abstract

The NGL Viewer (http://proteinformatics.charite.de/ngl) is a web application for the visualization of macromolecular structures. By fully adopting capabilities of modern web browsers, such as WebGL, for molecular graphics, the viewer can interactively display large molecular complexes and is also unaffected by the retirement of third-party plug-ins like Flash and Java Applets. Generally, the web application offers comprehensive molecular visualization through a graphical user interface so that life scientists can easily access and profit from available structural data. It supports common structural file-formats (e.g. PDB, mmCIF) and a variety of molecular representations (e.g. ‘cartoon, spacefill, licorice’). Moreover, the viewer can be embedded in other web sites to provide specialized visualizations of entries in structural databases or results of structure-related calculations.

## INTRODUCTION

Visualizing 3D molecular structures of biopolymers has become a common task in the life sciences (1,2). The depiction of, for instance, ligand-binding pockets or other details in macromolecular assemblies helps to elucidate the relationship between protein structure and function. The web in turn has long been a platform for providing access to 3D structural data itself and also to visualizations of it. Ongoing advances in web browser software facilitate this trend and allow the creation of rich visualization applications. Specifically, integration of browser-based visualizations simplifies access to results of structure-related calculations from web tools (3,4) and allows for quick depiction of entries in online structural databases (5–7). Hence, web browsers are extensively used for the visualization of molecular structures.

Until recently web browsers could not display 3D content without additional plug-ins. Therefore, programs like ‘Jmol’ (8) or the ‘OpenAstexViewer’ (9) needed to be embedded as Java Applets within a web page. However, the rise of the web as a platform for applications has resulted in more and more capabilities being added to the web browsers themselves. These include the HTML5 feature set and tremendous performance gains of JavaScript, the programming language available within web browsers. Eventually, this progress allowed the compilation of ‘Jmol’ into a pure JavaScript version called ‘JSmol’ (10), demonstrating the feasibility of browser-based molecular graphics without recourse to third-party plug-ins like Flash or Java Applets. In addition to molecular graphics, ‘JSmol’ provides the same features as ‘Jmol’, including user-scripts and sessions.

Another feature modern web browsers have is access to dedicated graphics hardware through the WebGL application programming interface (API). For desktop programs, shifting calculations to the graphics processor unit (GPU) has greatly improved rendering performance and quality (11). So by using the new capabilities of web browsers it is possible to provide hardware-accelerated graphics while coping with the increasing retirement of plug-ins. Some libraries have already been developed which leverage WebGL and can be embedded in web pages to provide molecular visualizations. Notable libraries include ‘pv’, as used in the SWISS-MODEL server (12), and ‘3Dmol.js’ (13). They provide developers with an API for the creation of molecular visualizations. Further, the ‘GLmol’-based (tinyurl.com/glmol) ‘iview’ employs GPU acceleration and provides an interface for viewing protein–ligand complexes (14).

Note that while WebGL is widely adopted by web browsers, there are still installations in which WebGL is not available, either due to missing software updates or older, unsupported hardware. To enable molecular graphics for these, ‘JSmol’ is an option, as it neither relies on WebGL for rendering nor requires a plug-in.

Here we introduce the ‘NGL Viewer’, which provides a rich graphical user interface (GUI) for customization of molecular scenes in addition to a developer API for embedding and controlling the viewer. Leveraging the features of modern web browsers, the ‘NGL Viewer’ can supply fast, hardware-accelerated molecular graphics and brings a familiar GUI to the web. The web application offers general-purpose molecular visualization and as it does not require the installation of specialized software it can simplify access 3D structural data for life scientists.

## IMPLEMENTATION

The ‘NGL Viewer’ web application is written mostly in JavaScript, with some code in HTML and CSS to create the GUI. Fast 3D graphics are enabled by GPU acceleration available through WebGL, a standard based on the OpenGL ES 2.0 API and built directly into the browser without the need for any plug-ins. All major web browsers support WebGL in their current versions through a 3D context for the HTML5 canvas element. Bindings to JavaScript in turn provide a flexible and low-level API to the GPU for web-based applications.

### Architecture

The source code is organized into task-specific modules: parsing and processing of molecular structures; transforming molecular structures into display representations such as spheres for atoms, sticks for bonds or tubes tracing a protein backbone; rendering of display representations on the GPU with WebGL; and, finally, creating an interactive GUI. A goal of this organization is a layered architecture with a clear separation of modules to facilitate re-use and refactoring in development. At the center of the implementation is the stage class. It supplies an instance of the viewer class for rendering and hosts component instances that contain, for example, the molecular structures for visualization. The component instances in turn host representation instances containing the geometry to be rendered. The GUI is decoupled from the stage class and reacts only to signals. For example, a stage instance emits a ‘component loaded’ signal to which the GUI reacts with the creation of an interface element for the loaded component. By that, no GUI-specific code is needed within the non-GUI parts. Generally, this architecture simplifies creation of custom GUIs and facilitates embedding the viewer into other web applications.

### Structures

Molecular structures are parsed and converted into an internal format based on the common structure-model-chain-residue-atom hierarchy of biomolecules. Parsers are currently implemented for the PDB, GRO and mmCIF file-formats. For large structures, each type of atomic data such as the coordinates or the element name is stored in a single JavaScript typed array. This saves memory compared to storing the data in properties of JavaScript objects for each atom individually.

### Visualization

To minimize time-consuming data exchange between CPU and GPU, the geometries of molecular representations are sent to the GPU in few but large arrays. The geometries in each of the arrays can then be rendered by a single draw call to the WebGL API.

**Figure 1.**
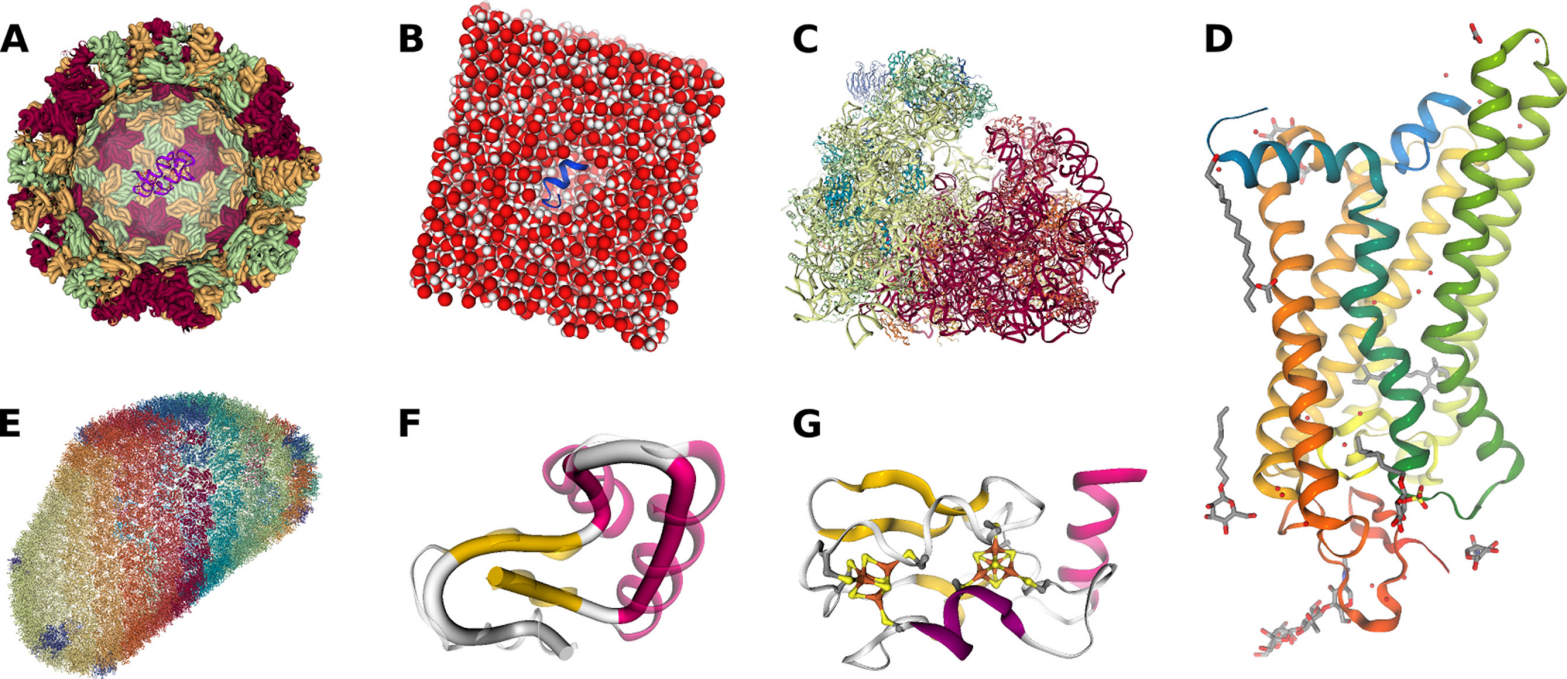
Gallery of molecular visualizations showing various representations of differently sized macromolecules. (**A**) Structure of the *Noro virus* capsid (PDB entry 1IHM) that forms the outer shell of the virus, colored by chainindex. The front of the capsid is clipped away and depicted inside the virus capsid is, for size comparison, the structure of a conserved retroviral RNA packaging element from the *Moloney murine leukemia virus* (PDB entry 2LIF). (**B**) System of a peptide derived from the C-terminus of ‘transducin’ (blue cartoon) surrounded by a box of water molecules (spacefill representation) in preparation for a molecular dynamics simulation. (**C**) Structure of a mammalian 80S ‘ribosome’ (PDB entry 4UJD) colored by chainindex. (**D**) Structure of light-activated ‘rhodopsin’ in complex with a peptide derived from the C-terminus of ‘transducin’ (PDB entry 3PQR), colored by residueindex. (**E**) *HIV-1* capsid structure (PDB entry 3J3Y) showing the backbone colored by chainindex. (**F**) Example usage of the rope representation (solid tube) to display the protein fold in a more abstract way as compared to the cartoon representation (translucent). The structure shown is that of ‘crambin’ (PDB entry 1CRN) colored by secondary structure with magenta alpha-helices and yellow beta-strands. (**G**) The secondary structure of ‘ferredoxin’ (PDB entry 1BLU) and its two [4Fe–4S] clusters highlighted with the ‘HyperBall’ representation.

**Figure 2.**
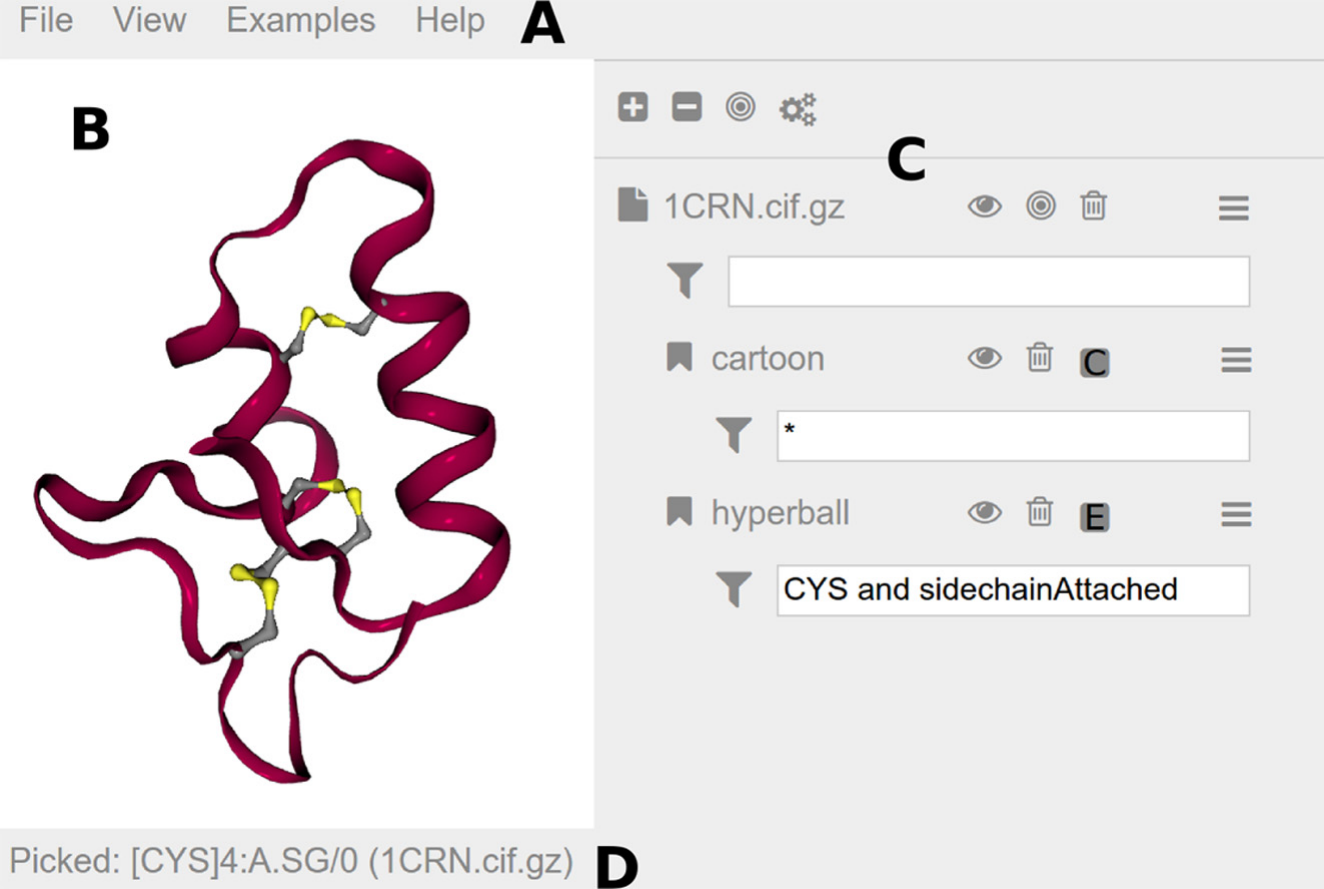
Screenshot of the ‘NGL Viewer’ GUI, magnified for clarity. A more detailed description of the GUI can be found in the online documentation. (**A**) The menu bar at the top provides access to general commands. The ‘File’ menu includes commands to load files or export images. Buttons to change the theme or to go fullscreen are in the ‘View’ menu. The ‘Examples’ menu includes various possible use cases. Help-related items like a preferences section and a link to the documentation can be found in the ‘Help’ menu. (**B**) Molecular visualizations are rendered to the viewport at the center. Here the structure of ‘crambin’ (PDB entry 1CRN) is shown highlighting the three disulfide bridges. (**C**) The sidebar hosts an interface element for each loaded structure and added representations. A number of buttons are available to quickly access commands: hide/show (eye icon), center (bulls eye icon), delete (trash bin icon) and parameters menu (stacked bars icon). Input fields for atom selections (funnel icon) restrict the display of representations. Here the ‘hyperball’ representation is limited to CYS and sidechainAttached. (**D**) The status bar at the bottom notes the last picked atom, in this case the ‘SG’ atom in the ‘cysteine’ with residue number ‘4’ in chain ‘A’ of model ‘0’.

For the rendering of spheres and cylinders, ray-casted impostors are used to greatly reduce the geometric complexity and enable display of very large macromolecules with sphere- and cylinder-based representations (15,16). For older hardware that does not support the required EXT_frag_depth WebGL extension, spheres and cylinders are rendered as meshes of triangles forming the geometric primitives. Ray-casting is also used to provide an implementation of the ‘HyperBalls’ representation (17) in which atoms are smoothly connected by an elliptic hyperboloid (Figures 1G and 2B).

The line and point primitives available in the WebGL API provide visually simple but very fast representations for huge macromolecules or when older hardware is used. All remaining representations such as a tube tracing the backbone atoms are rendered as triangle meshes.

The ‘three.js’ library (threejs.org) is used to create and render the scene containing the geometries of molecular representations. The library also provides the camera implementations and mouse controls.

### Interaction

Picking atoms with pixel-level accuracy is implemented GPU-based by rendering the scene off screen with colors that uniquely identify all pickable elements. Upon clicking on the canvas, the color of the pixel under the mouse pointer is read identifying the corresponding element, for instance a specific atom.

A concise language for selecting parts of molecular structures from models and chains to atoms was created, with a syntax inspired by the atom expressions available in the scripting language of ‘Jmol/JSmol’. Strings written in the selection language are parsed and transformed into testing functions that can be applied at all levels of the structure-model-chain-residue-atom hierarchy to efficiently determine if an entity belongs to the selection or not. For example, the selection string ALA and .CA breaks down to tests for ‘alanine’ residues and ‘C-alpha’ atoms. All atoms for which both tests evaluate to true are selected.

## WEB APPLICATION

The ‘NGL Viewer’ web application enables interactive display of large macromolecules and allows complex manipulations of the visualization through a GUI. Structures in PDB, mmCIF or GRO format can be loaded into the application from local and remote sources. For instance, local files can be loaded via drag'n’drop and PDB entries are retrieved from the RCSB archive (www.rcsb.org) by their ID. Moreover, compressed files in ‘gz’ or ‘zip’ format are automatically unpacked upon loading.

Structures loaded into the viewer are displayed using a variety of representations that can be combined to create complex molecular views (Figure 1). Multiple representation types are supported, including cylinders and spheres for bonds and atoms (‘spacefill, ball+stick, licorice, line, point’) as well as secondary structure depictions based on backbone atoms (‘cartoon, tube, ribbon, trace, backbone, rocket’). The appearance of the representations is tunable by parameters to create unique styles and also to trade quality for performance. Most representations have a color and a radius parameter that can use data from the underlying structure. For instance, a representation can be colored uniformly or according to the element, residue or secondary structure type of the atoms from which the representation is derived. The size of representation objects, such as sphere and cylinder radii in a ‘ball+stick’ representation, is set similarly. The GUI provides controls to add new representations to a structure and to change the parameters of existing ones (Figure 2C). Clicking on a representation prints the full name of the entity (i.e. atom, residue, chain, model, structure) it belongs to (Figure 2D).

Display of representations is easily limited to specific atoms, residues or chains via a concise selection language. This allows detailed control of which parts of a biomolecule are shown to highlight important aspects or are not shown for clarity (Figure 2B and C). For example, the side-chain and C-alpha atoms plus the backbone nitrogen in the case of proline are selected with side-chain or .CA or (PRO and .N). For convenience, the shorthand sidechainAttached is available. A detailed description of the selection language can be found in the online documentation.

Large systems of biomolecules are often available as coarse-grained structures where only the backbone atoms are resolved. Here, visualization of coarse-grained structures is supported by special treatment when calculating backbone and side-chain bonds as well as by automatic alpha-helix detection. Thus, representations such as ‘cartoon or ribbon’ are available for coarse-grained structures despite their missing atoms. Also provided is a ‘rope’-like protein fold abstraction especially suitable for coarse-grained structures. In this representation a tube follows a local axis similar to what is done in ‘Bendix’ (18).

Structure files from the PDB include data on biologically relevant assemblies and provide information about crystallographic symmetries. Display of the corresponding assemblies can be controlled via the parameter menu of structures and representations (Figure 2C).

The molecular views created can be exported as anti-aliased high-resolution images for use elsewhere.

### Embedding

The viewer can be embedded into other websites by including a single JavaScript file and then calling API methods to create a stage instance that allows loading and subsequent manipulation of molecular structures. Instructions and examples for embedding and controlling the viewer are available in the online documentation along with a description of the API methods.

## CONCLUSION

By leveraging modern web technologies such as WebGL, the ‘NGL Viewer’ provides scalable molecular graphics allowing the display of large macromolecular assemblies including ribosomes and virus capsids (Figure 1). It is accessible through a user-friendly GUI (Figure 2) but can also be embedded in other websites as a library. The viewer supports common structure file formats, including PDB and mmCIF. To create complex molecular views, a wide array of molecular representations is available, including the atomistic display of atoms and bonds as well as secondary structure depictions based on backbone atoms. Moreover, we implemented state-of-the-art representations known from desktop applications, including ray-casted primitives, ‘HyperBalls’ and a ‘Bendix-like’ representation (15–18).

The web application is free and open to all users and there is no log-in requirement. Moreover, the full source code is available at https://github.com/arose/ngl/ under an open-source license. Extensive documentation can be found at http://proteinformatics.charite.de/ngl/doc.
